# Supplementary figures and images for: Manipulation of light quality is an effective tool to regulate photosynthetic capacity and fruit antioxidant properties of Solanum lycopersicum L. cv. ‘Microtom’ in a controlled environment
Source: PeerJ. 2022 Jul 1;10:e13677. doi: 10.7717/peerj.13677 (PMC9252183; doi:10.7717/peerj.13677)

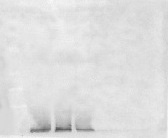

Supplement: Supplemental Information 1 [file peerj-10-13677-s001.jpg]

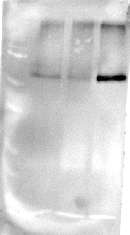

Supplement: Supplemental Information 2 [file peerj-10-13677-s002.jpg]

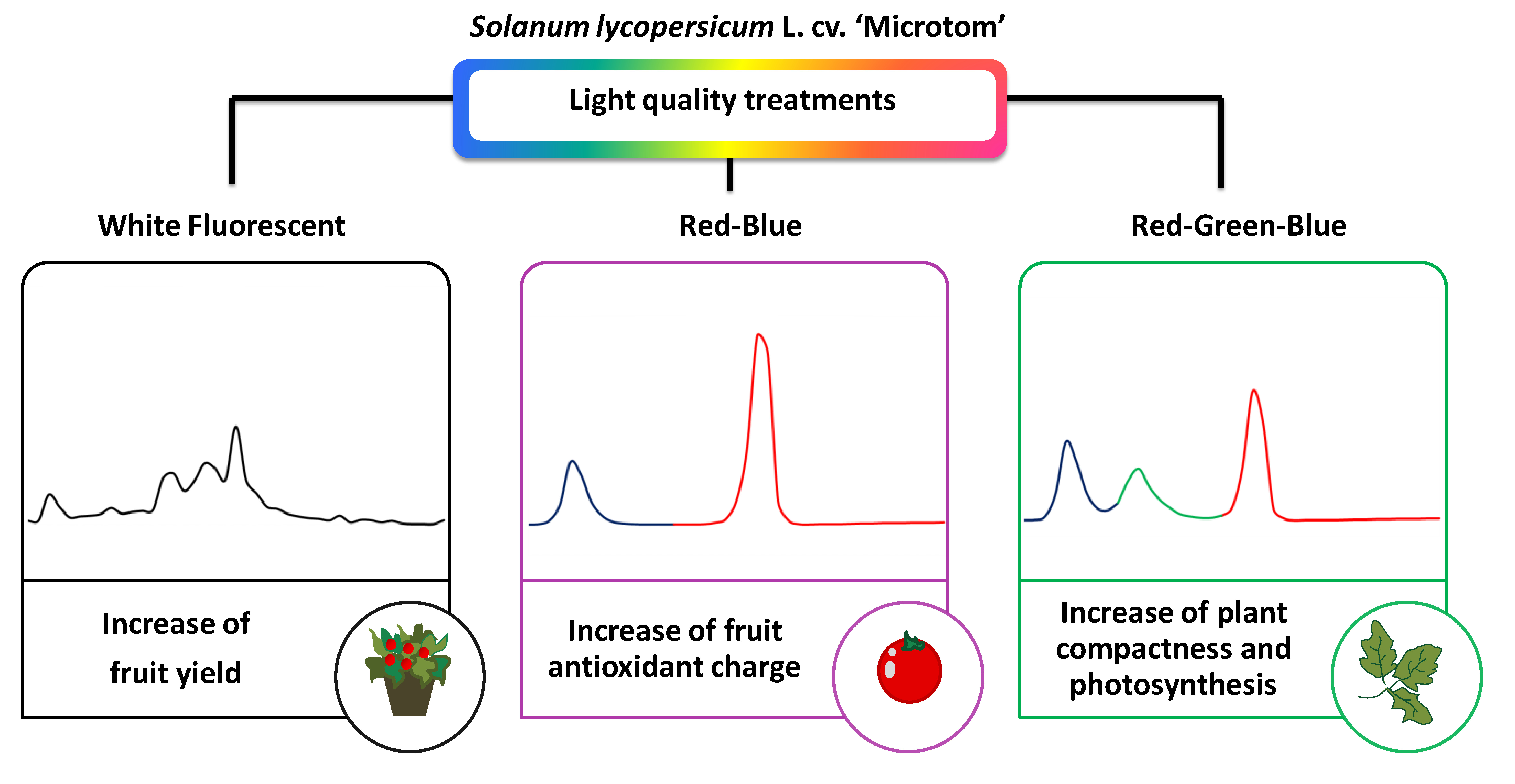

Supplement: Supplemental Information 4 [file peerj-10-13677-s004.png]
